# Protein-Carbohydrate Interactions Studied by NMR: From Molecular Recognition to Drug Design

**DOI:** 10.2174/138920312804871175

**Published:** 2012-12

**Authors:** María del Carmen Fernández-Alonso, Dolores Díaz, Manuel Álvaro Berbis, Filipa Marcelo, Javier Cañada, Jesús Jiménez-Barbero

**Affiliations:** Centro de Investigaciones Biológicas-CSIC. Calle Ramiro de Maeztu, 9. 28040. Madrid, Spain

**Keywords:** NMR, molecular modeling, protein-ligand interactions, drug targeting.

## Abstract

Diseases that result from infection are, in general, a consequence of specific interactions between a pathogenic organism and the cells. The study of host-pathogen interactions has provided insights for the design of drugs with therapeutic properties. One area that has proved to be promising for such studies is the constituted by carbohydrates which participate in biological processes of paramount importance. On the one hand, carbohydrates have shown to be information carriers with similar, if not higher, importance than traditionally considered carriers as amino acids and nucleic acids. On the other hand, the knowledge on molecular recognition of sugars by lectins and other carbohydrate-binding proteins has been employed for the development of new biomedical strategies. Biophysical techniques such as X-Ray crystallography and NMR spectroscopy lead currently the investigation on this field. In this review, a description of traditional and novel NMR methodologies employed in the study of sugar-protein interactions is briefly presented in combination with a palette of NMR-based studies related to biologically and/or pharmaceutically relevant applications.

## 1 INTRODUCTION

The last decades have witnessed the focus on nucleic acids, proteins and their mutual interplay as the pre-eminent entities responsible for the storage and transmission of biological information in living organisms. Although underappreciated in the genomic and post-genomic eras, carbohydrates are now emerging as central players in the translation of encoded information into functional activity, thanks to the acknowledgement of their enormous coding capacity and the increasing realization of their importance in protein-mediated recognition events leading to biological responses. Carbohydrate messages encoded in free oligosaccharides, glycoproteins and glycolipids are interpreted by a plethora of lectins and other glycan-binding receptors, which translate them into cellular activity. The involvement of protein-carbohydrate interactions in crucial processes with deep implications in human health and disease such as growth regulation, tumor cell adhesion, cell migration or host-pathogen recognition has raised interest towards the comprehension of such recognition phenomena from multiple points of view. In recent times, the development of methodologies such as X-ray diffraction and nuclear magnetic resonance spectroscopy have permitted to gain insight into the geometry of protein-carbohydrate interactions at the atomic level. At the same time, such structural information has provided starting points for the clinical targeting of protein-carbohydrate interactions.

The present review summarizes the achievements, ongoing research and current challenges of the understanding of sugar-protein interactions and their implications for human physiology and pathology, mainly focusing on the contributions of our group to the field.

## 2 CARBOHYDRATE-BINDING PROTEINS. THE NATURE OF THE CARBOHYDRATE-PROTEIN INTERACTION

The notion of carbohydrates being specifically recognized by proteins dates back to the ‘lock-and-key’ principle used by Emil Fisher as early as 1894 [1] to illustrate the complementarity between a glucoside and an enzyme that permits the one to fit into the other. The advent of modern techniques devoted to the structural elucidation of biomolecules, have permitted to gain detailed insight into the mechanisms governing the phenomenon of molecular recognition, providing a thorough understanding of the physicochemical bases underlying the receptor-ligand interaction.

Carbohydrates interact with partners belonging to a number of protein families including lectins, antibodies, sugar transporters and enzymes, such as glycosyl transferases. The latter catalyze the synthesis of activated sugar donors, as well as the transfer of glycosyl residues from these donors to other carbohydrates or aglycans, and thus they are responsible for the genesis of the whole cellular glycan repertoire. For their part, antibodies raised against carbohydrate epitopes are generated by the immune system and play a role in the defense against pathogens and cancer cells.

In contrast to antibodies, lectins are not products of the immune system and they display structural diversity. Furthermore, and unlike enzymes, they lack catalytic activity. Etymologically from the Latin *legere *[2], meaning, among other things, to select, lectins recognize and bind selectively and specifically to carbohydrate epitopes of glycoproteins, glycolipids and free oligosaccharides without modifying them.

**Fig. (1):**
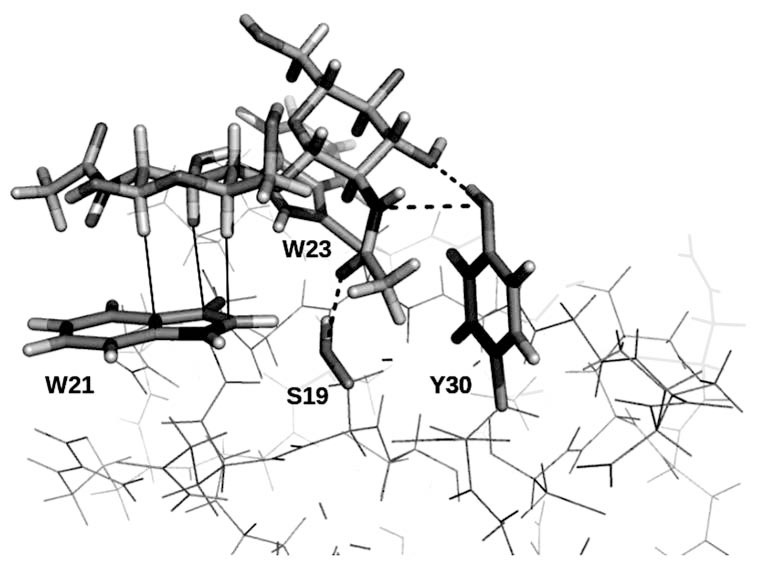
Protein-carbohydrate interactions governing the recognition of chitobiose by hevein, as determined by NMR.[4] Dashed lines represent hydrogen bonds established between the sugar and polar residues of the protein (S19 and Y30). Solid lines indicate CH-π interactions with the W21 indole ring. Stacking contacts established with W23 are here omitted for clarity.

First discovered in plants, lectins are now known in all kingdoms of life. Many plant lectins are involved in defense against parasites or predators, being sometimes secreted in large quantities as highly toxic agents, such as ricin. Thanks to the easy disposal and purification of legume lectins, many of which are available on a commercial basis, plant lectins find themselves among the better characterized systems. We have profusely employed hevein as a model system to study protein-carbohydrate interactions [3]. The hevein domain is found in many legume lectins, such as hevein itself, its natural cognate pseudohevein, the wheat germ agglutinin (WGA), the *Urtica dioica* agglutinin (UDA) or the Ac-AMP antimicrobial peptides. These proteins have specificity towards β(1-4) linked N-acetylglucosamine (GlcNAc) glycans, notably chitin, a polysaccharide found in fungal cell walls and in the exoskeleton of insects and other invertebrate parasites. Being a small protein of only 43 residues, hevein constitutes a very amenable system to work with. Using NMR spectroscopy, we have obtained detailed information on affinity and binding geometry for several hevein-(GlcNAc)_n_ complexes, and we provided the first structure of a protein-ligand complex solved by NMR (a detail of this structure is shown in Fig. **1**).[4]

### 2.1 The Nature of the Carbohydrate-Protein Interaction

In general, glycans bind at domains defined by shallow pockets on the hydrophilic surface of lectins and other proteins. As a consequence, affinity regimes of monovalent interactions between lectins and their ligands tend to be rather weak. Multivalency is thus a key concept in lectinology, as it permits not only the affinity of the interaction between proteins and glycans to be significantly enhanced, but also other phenomena to emerge, such as agglutination.[5]

Overall, sugar binding to lectins is made possible via a number of attractive forces including hydrophilic and hydrophobic interactions. Despite the phylogenetical ubiquity and structural diversity of carbohydrate-binding proteins, the signature of the protein-carbohydrate interaction can be seen in different protein families and all throughout the evolutionary tree.

#### 2.1.1 Hydrogen Bonding

The establishment of hydrogen bonds between sugars and their receptors constitutes perhaps the most evident lectin-carbohydrate bonding potential due to the existence of numerous -OH groups in all saccharides (Fig. **1**). Furthermore, hydrogen bonding is made possible not only thanks to the hydroxyl groups, but also to the presence of amine and carboxyl groups of many substituted carbohydrates. 

Hydroxyl groups may participate in hydrogen bonds both as donors and as acceptors, by means of their lone ion pair. In some instances, the same -OH group acts simultaneously as donor and acceptor, a characteristic phenomenon in sugar-protein interactions which is known as “cooperative hydrogen bonding”.

Sugar hydroxyl groups establish hydrogen bonding contacts with the side chains of polar residues, most often aspartic and glutamic acid, asparagine, glutamine, arginine and serine, as well as backbone amine and carbonyl groups. A characteristic feature of carbohydrate-protein interactions commonly seen not only in lectins, but also in enzymes, are bidentate hydrogen bonds established between two adjacent hydroxyl groups of a sugar and both carboxylate oxygens of either aspartic or glutamic acids. [5]

Of course, hydrogen bonding not only contributes to affinity but also to selectivity, being in many cases the characteristic stereochemical arrangement of hydroxyl groups what gives a protein its specificity towards a given sugar type. We have studied the implications of hydrogen bonding on selectivity by approaching the phenomenon from both perspectives, i.e. those of the lectin and of the sugar, i.e. by studying the effect of mutation of key residues of the former [6], and using engineered sugars ‘deleterious’ for hydroxyl groups via the synthesis of monodeoxy [7] and fluoro-monodeoxy derivatives [8, 9] of natural lectin ligands.

#### 2.1.2 Apolar Interactions

In some sugars, the clustering of three or more adjacent C-H groups caused by the characteristic steric disposition of hydroxyl groups creates hydrophobic patches on the sugar surface that can establish apolar interactions with hydrophobic epitopes in proteins, most notably the aromatic rings of Trp, Tyr and Phe residues. Although the fine details of the nature of such interactions are currently subject to investigation, it is thought that attractive forces are due to both an entropic contribution, arising from the mutual shielding of both apolar surfaces from the bulk water, and the enthalpic contribution of non-conventional hydrogen bonds established between the partially positively charged C-H groups and the quadrupole created by the π-system of the aromatic ring (Fig. **1**).[3]

We have contributed to the understanding of CH-π interactions using a multidisciplinary approach combining NMR spectroscopy and molecular mechanics calculations. Using simple models involving monosaccharides and small aromatic compounds, such as free aromatic amino acids, we were able to detect and quantify the magnitude of their interaction in water by simple NMR experiments.[10] In addition, we provided experimental evidence for the hydrophobic component to the interactions, finding that they were absent in aprotic solvents.[11] Theoretical results obtained in our laboratory also indicated that the carbohydrate-aromatic interaction is enthalpically stabilized by weak electro-attractive forces between the sugar hydrogens and the aromatic ring,[12] a hypothesis that we confirmed experimentally by monitoring the loss of affinity upon deactivation of the aromatic rings by the attachment of fluorine atoms to aromatic residues of Ac-AMP-like peptides.[13]

#### 2.1.3 Other Interactions

Further forces involved in the recognition of some carbohydrates by their protein receptors include salt bonds between electrically charged residues of some saccharides, such as sialic acid, and protein residues of opposite charge. Another unusual interaction involves the coordination of a divalent cation bridging certain sugar hydroxyls and negatively charged aspartates or glutamates. Such interaction is displayed by proteins belonging to the C-type family of lectins, which require the presence of Ca^2+^ ions to recognize their carbohydrate ligands.

#### 2.1.4 Distortion of Sugar Rings

Attending to structural and theoretical studies on the molecular recognition of oligosaccharides and their analogues by glycosidase and glycosyltransferase enzymes, distortion of the ground-state conformation of the saccharide ligand has been observed on numerous occasions.[14] The attained degree of distortion may resemble the proposed oxocarbonium-type transition state of glycoside hydrolysis.[15,16] With the idea of understanding the behavior of such systems, we have considered the determination of the energy associated with the deformation in non-substituted rings as a first step. This knowledge is of paramount importance to comprehend and predict the outcome of the recognition process and to rationally design inhibitors of biomedical processes in which glycosidases and glycosyltransferases are involved. The inversion process of cyclic compounds is the cornerstone of conformational analysis and in our group we have performed several theoretical studies in that direction, by using as model systems non-substituted six-membered rings (cyclohexane, oxane, thiane) as well as mono-methyl-substituted versions of them.[17] Those studies have allowed the rigorous determination of complete conformational inversion processes for these rings as well as a precise determination of the energetic barriers that need to be overcome for the conversion between the different conformers.

## 3 CHARACTERIZATION OF PROTEIN-CARBOHYDRATE INTERACTIONS BY NMR SPECTROSCOPY

A detailed description of the conformational preferences of carbohydrates, both free and bound to proteins, is crucial for a better understanding of the sugar recognition process. Many biophysical and computational techniques have been employed to gain knowledge about this from different perspectives. X-ray analysis [18], NMR spectroscopy [19], molecular modeling [20], mass spectrometry [21], surface plasmon resonance [22] and calorimetry [23], among others, have been used for determining key structural aspects for the formation of carbohydrate-protein complexes. 

From the above mentioned techniques, X-ray crystallography as well as NMR spectroscopy (alone or in combination with computational calculations) have become, undoubtedly, the main sources of experimental information on sugar-receptor complexes. On the one hand, numerous free and complexed biomolecules have been characterized by X-ray crystallography and the resulting structures have been taken as static pictures of stabilized complexes. On the other hand, the characteristic flexibility of carbohydrates that may be critical in the recognition process is not fully reflected in a crystal structure and combined NMR and computational studies offer a variety of approaches to highlight different features of the sugar binding process. 

The effect of sugar binding can be traced by the perturbations observed on characteristic NMR parameters (e.g. chemical shift, relaxation rates, line width, signal intensity, cross-relaxation) of either protein or carbohydrate giving the technique a wide applicability to a range of receptors and oligosaccharides types. The conformational flexibility of carbohydrates, especially in the glycosidic torsion angles and lateral chains as well as different (possible) ring shapes, precludes, in general, a straightforward analysis of NMR data and additional computer simulations are usually needed to get a better picture of the dynamic process associated to the binding. 

Most carbohydrate receptors are large molecules (MW higher than 30 kDa) with intricate NMR spectra from which binding information is difficult to extract. Carbohydrate spectra are, in comparison, more manageable and, currently, most of the NMR-based methods to study protein-carbohydrate interactions are fairly focused on the observation of carbohydrate resonances. Herein, we discuss representative methods and examples of both protein- and carbohydrate-detected NMR-based methods to scrutinize the factors that govern sugar-receptor interactions. 

### 3.1 Binding Modes and Molecular Modeling of Carbohydrate-Protein Complexes

Prior to MD analysis, docking methods are very often used to predict the orientation of the carbohydrate in the binding site. The docking algorithms (Autodock [24], GLIDE [25], Dock [26]) take into account the orientations of pendent groups (e.g. hydroxyl groups and the possible formation of a hydrogen bond network that stabilizes the complex) as well as the conformational behavior of the glycosidic linkages for oligosaccharides. The structure with the best scoring function resulting from docking calculations is normally used for subsequent MD simulations. 

For the specific case of sugar-receptor complexes, several force fields including energy parameters for describing energy minimization or MD simulations of sugar-protein complexes are available (AMBER/GLYCAM [27], TRIPOS [28], GROMACS [29], CHARMM [30], OPLS [31] and MM3 [32]). Current technology and software development allow for MD simulations of sugar-protein complexes rising up to hundreds of nanoseconds, which are long enough to sample the conformational space of the system. 

More detailed information on the 3D structure and dynamics of sugars in both free and bound state can be pictured by complementing the NMR experimental data with molecular modeling and MD simulations. Thus, averaged NMR experimental parameters can be deconvoluted by MD analysis to evaluate the weight of each of the structural factors favoring stability of lectin-carbohydrate complexes. Certain aspects regarding the interaction between sugars and proteins remain unrevealed because of the intrinsic limitations of molecular mechanic calculations. Therefore, hybrid methods combining *ab initio* quantum mechanics and molecular mechanics (QM/MM) computations combined with all-atom molecular dynamics (MD) simulations are being employed for the study of protein-sugar interactions from an atomic-electronic point of view.

### 3.2 Protein-Detected NMR Based Methods

For protein-detected NMR studies of small carbohydrate binding proteins, chemical shift or line-width variations of specific proton resonance(s) (the so-called reporter/probe nuclei), as induced by the presence of the sugar, may suffice for proper monitoring of the binding event. In contrast, for medium-large proteins, isotope labeling (^13^C, ^15^N) is imperative unless the binding event is monitored from the ligand perspective by NMR means (see below).

#### 3.2.1 Model Proteins: ^1^H Chemical Shift Mapping 

Hevein, a very well-characterized small lectin that specifically recognizes GlcNAc and GlcNAc-containing carbohydrates (e.g. chitin), has been exhaustively used as a reference to better understand the individual contributions of the numerous factors that favor carbohydrate-protein interactions. Thus, classical ^1^H-NMR titration experiments have allowed for precise determination of binding affinity of hevein to a variety of GlcNAc-based oligosaccharides (i.e. association constants, K_a_) by correlating chemical shift variation of selected proton resonances of the protein at increasing ligand concentrations. The titration ends with complete saturation of the binding site and subsequent interpretation of the NMR data has demonstrated that the binding process is enthalpy-driven. [4] The same strategy has been applied on modified heveins which retain native-like carbohydrate-binding properties. These studies revealed that, besides hydrogen bonding and van der Waals forces, the presence of aromatic residues in the binding site, which are prone to produce stacking (the so-called CH-π interactions), is significant for both sugar binding affinity and stability of carbohydrate-protein complexes. [6,33] Furthermore, the role of carbohydrate-aromatic interactions has also been explored for mutant AcAMP2-like peptides in which key interacting residues have been replaced by non-natural aromatic residues and demonstrated that both size and aromaticity correlate to association constant and binding enthalpy values.[13]

#### 3.2.2 Isotope Labeling (HSQC) and Relaxation 

Isotope labeling enables monitoring of sugar binding by observation of chemical shift and/or relaxation rate variations on detected ^1^H/^13^C/^15^N signals, associated to ligand binding. As an example of a complete NMR study in such a scale, the binding of a heparin-like hexasaccharide to the acidic fibroblast growth factor (FGF-1) is presented. An initial investigation on the binding site of FGF-1 was carried out by chemical shift perturbation analysis of the FGF-1 ^1^H and ^15^N NMR resonances upon addition of the ligand (i.e. 2D ^1^H-^15^N HSQC). Comparison of the chemical shifts of the protein in the absence and in the presence of the carbohydrate showed that signals corresponding to nuclei located inside the binding pocket were the most shifted upon sugar binding, allowing for the identification of the protein binding epitope. Moreover, this result was in agreement with the X-ray structures of FGF in complex with sulfated glycosaminoglycans. [34,35] Besides chemical shift perturbations, determination of the dynamic properties (or relaxation rates) of receptor residues for the free and bound states also demonstrated that there is a variety of internal motions and chemical exchange in the free state, particularly at the heparin binding site, which decreases considerably upon hexasaccharide binding, in both the fast and slow dynamic timescales. These observations suggest that such motions may be involved in the regulation of heparin binding and subsequent activation of FGF-1.[36]

In this context, modification of the relaxation properties of the sugar/galectin-3 complex by inclusion of a paramagnetic tag covalently bound to the sugar molecule (spin-labeled analogues of natural ligands, e.g. TEMPO (2,2,6,6-tetramethylpiperidin-1-oxyl)) causes perturbation of relaxation properties of the closest ^1^H,^15^N galectin-3 amide signals, which were identified via ^15^N HSQC. The intensity of the amide cross-peaks diminished in a distance-dependent manner to the paramagnetic center and the authors proposed a model to convert intensity measurements into intermolecular distances, which allowed for a more precise delineation of the protein binding epitope. [37] 

### 3.3 Carbohydrate-Detected NMR Based Methods

The earliest NMR methods for determination of ligand-protein complex were based in the measurement of T_1_/T_2_ relaxation rates of ligand protons, as those are related to molecular motion, which changes drastically upon binding to a macromolecular receptor. Thus, the observation of selective broadening of certain resonances of the ligand (some of which can even disappear) indicates that they are in close contact with the protein and, as a consequence, their T_1_/T_2_ relaxation times are shortened. 

The binding of a C-glycosyl analogue of N-acetyl-lactosamine glycomimetics to viscumin (mistletoe lectin, VAA) and human galectin-1 (hGal-1) have been followed by the detection of signal broadening due to the shortening in T_2_ relaxation times. An additional monitoring of T_1_ values demonstrated that the selective T_1_ value of H1-Gal strongly decreases when passing from the free to the bound state, again indicating ligand binding. [38]

New NMR strategies for studying sugar-protein complexes are currently based on exploring the use of paramagnetic probes to obtain structural information on both the sugar and protein epitopes. These novel approaches exploit the concomitant effects of both line width broadening due to paramagnetic relaxation enhancement (PRE) as well as the shift of signals or pseudo-contact shifts (PCS), since they are indicative of their relative distance to the metal. [39] Additionally, proteins containing adequate paramagnetic tags that contribute to a spontaneous alignment of the macromolecule with the external magnetic field can be also used to reveal residual dipolar couplings (RDCs). [40]

Recently, paramagnetism-based NMR constraints, including pseudo-contact shifts (PCS) and field-induced residual dipolar couplings (RDCs) have been used to study the Galectin-3-lactose system. By attaching a lanthanide-binding peptide to the Galectin-3-carbohydrate-binding domain, the authors observed an agreement between their experimental results (NOEs, PCSs, RDCs) and the crystal structure of a Galectin-3-N-acetyl-lactosamine complex. [41]

#### 3.3.1 Diffusion Spectroscopy 

Measurement of the diffusion coefficient of sugars (free and bound) by NMR diffusion ordered spectroscopy (DOSY) have been performed for the detection of carbohydrate interactions in solution.[42] The translational diffusion coefficient is characteristic for each carbohydrate and comprises information about both, its molecular size and shape. Upon binding, the hydrodynamic radius of the sugar is apparently increased and, thus, its diffusion coefficient is reduced. 

In particular, changes in the diffusion coefficients of free and bound sugars have been monitored to study the multivalency of chitin binding to hevein domains.[43] This technique has been successfully used for screening of chemical mixtures [44], detection of ligand-protein complexes [45] and analysis of small metabolites in biofluids [46].

This approach has been extended for determining the use of sugar-modified gold nanoparticles acting as receptor and the same carbohydrate as the ligand. A significant change in the diffusion coefficient of the glyconanoparticle is detected only in the presence of calcium ions, as they are crucial for the sugar-nanoparticle interaction. This research extended the employment of DOSY methods to detect carbohydrate interactions besides those frequently encountered in the cyclodextrin field and to monitor molecular size in natural and artificial carbohydrate polymers and glycodendrimers.[47]

#### 3.3.2 NOE-Based Methods

NOE-based methods are nowadays the most powerful techniques for elucidation of key aspects of the sugar-receptor interaction from the ligand’s perspective. They are, however, only applicable when the off-rate of the dissociation process of the ligand from the bound to the free state is fast. Otherwise, alternative methods must be applied. As an example, for the slow exchange heparin-FGF-1 system, ^13^C-labelled FGF-1 has been employed to determine the bound conformation of the ligand by means of half-filtered NOESY experiments. In the resulting NOESY spectrum, the peaks of protons attached to ^13^C are removed and only sugar cross-peaks are shown. [48]

##### 3.3.2.1 Transferred NOE

**Fig. (2):**
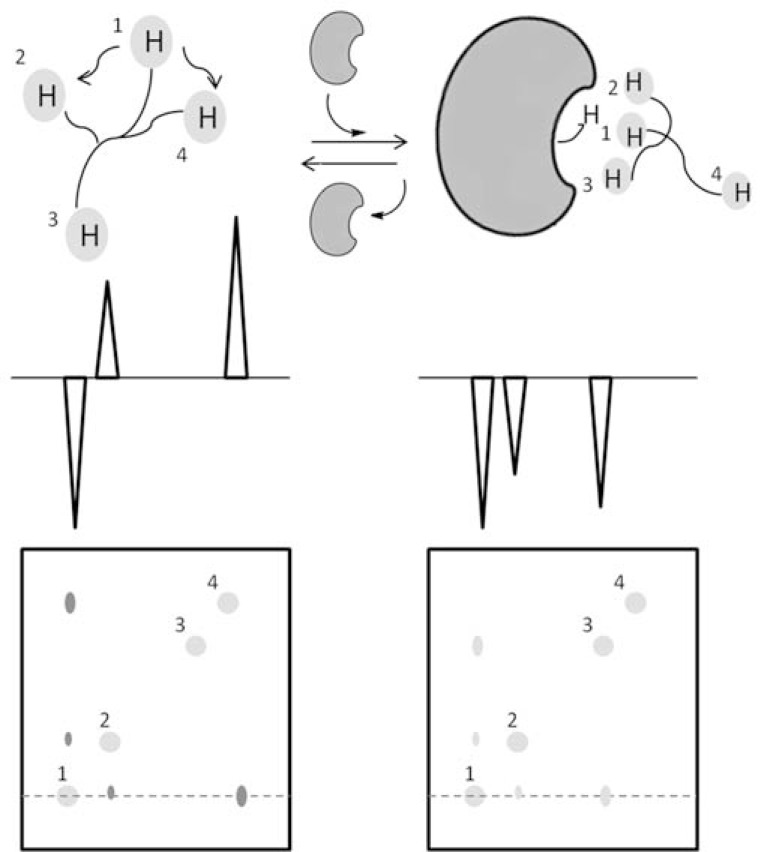
Schematic representation of the TR-NOE experiment.

From the structural viewpoint, intramolecular NOEs are decisive in determining conformational preferences of carbohydrates in solution (in both free and bound states). According to their molecular weight, sugars in the free state have, in general, characteristic short tumbling times (τc) and exhibit, normally, positive NOEs. Upon binding, carbohydrates behave as part of the sugar-protein complex and show negative NOEs as if the tumbling time of the sugar would have been drastically increased as a consequence of the binding event (a new but virtual molecular weight). See (Fig. **2**) for a schematic representation. This behavior is the so-called transferred NOE (trNOE) and a simple comparison between the NOE spectra of the sugar both, free and in the presence of the protein, would suffice to prove whether interaction takes place. In addition, the analysis of intramolecular NOEs in the trNOE spectrum is useful for determining sugar conformation in the bound state (intramolecular NOEs), while intermolecular NOEs (sugar-protein), if observable and identifiable, indicate the orientation of the sugar with respect to the binding pocket. As mentioned above, for slow off-rate on the relaxation timescale (turnover of a few times per second or less), the trNOE effect will not be detectable due to relaxation before the carbohydrate dissociates from the receptor. 

Interestingly, for the interaction between hevein and the Asn-linked Man (GlcNAc)_2_, only part of the ligand resonances showed trNOE as a consequence of the binding (negative cross peaks, long correlation times) while the rest (Asn) of the molecule exhibited the opposite effect. This result indicated that the fragment of the ligand that keeps its flexibility interacts weakly with the protein. [49]

##### 3.3.2.2 Methods Based on (Direct or Indirect) Transfer of Receptor-to-Ligand Magnetization 

The most successful NMR-based methods for studying ligand-protein interactions rely on magnetization transfer from the receptor to the ligand. The experiments described below are, undoubtedly, very well suited for determining affinity properties of either a single ligand or a mixture of ligands towards a receptor. The only limitation of this group of methods is that the ligand must be in fast exchange between free and bound states on the NMR time-scale. 

**Fig. (3):**
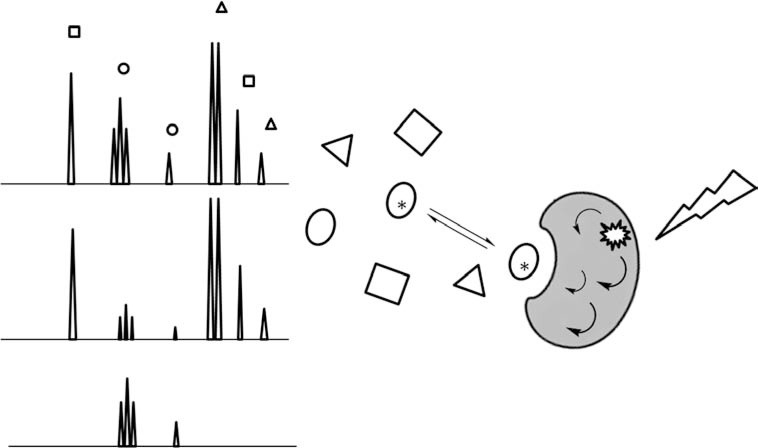
Schematic representation of the STD experiment.

Saturation Transfer Difference NMR spectroscopy (STD-NMR) STD is currently the most versatile and used NMR-based method for detection of protein-ligand interactions. As shown in (Fig. **3**), the experiment is based on the complete saturation of protein resonances and the subsequent transfer of magnetization via intermolecular ^1^H-^1^H cross-relaxation from the receptor to the closest ligand molecule located in the binding site.[50] The outcome of the experiment is a spectrum that displays only the ligand signals that were in direct contact to the saturated protein resonances and they are identified as the ligand-epitope. A closer look at the intensities of the resulting STD signals allows for quantification of the individual contributions of key structural elements.[ 51] The ligand topology can be mapped by comparing theoretical STD-NMR intensities from a simulated carbohydrate-protein complex and the experimental data (i.e. CORCEMA-ST) [52] 

Among other advantages, the experiment requires only small amounts of non-labeled protein and allows for simultaneous screening of a large number of potential ligands against the same target receptor. Thus, a rough estimation of the binding affinity properties of the components of a mixture of ligands can be achieved, as ligands can be ranked according to their STD-NMR intensities. [53]

The STD experiment is very sensitive for weak-medium binders, as it is the case for most carbohydrate-lectin systems, but its major drawback is that it does not discriminate between tight-binders and non-binders. In such a case, additional STD experiments in which a weak ligand (reporter ligand) can compete for the occupancy of the binding site should be performed. [53]

Typically, transfer of magnetization occurs from the receptor protons to the ligand protons (^1^H- ^1^H) but, recently, the presence of other NMR-active nuclei (i.e. ^19^F) has been also exploited as an additional STD-based reporter atom for detection of ligand binding (^1^H-^19^F). Thus, recent studies involving lectins and a series of potential fluorine-containing inhibitors (i.e. fluorinated carbohydrate derivatives) have been investigated by means of STD, through magnetization pumping from the lectin protons to ligand fluorines. [54] Furthermore, the complexity of the final STD spectrum becomes significantly reduced and few ^19^F signals have to be considered. 

When overlapping of signals makes the analysis of STD data difficult, as it usually occurs with carbohydrates, additional NMR experiments that combine STD-NMR with other standard NMR experiments have been designed for better identification of the binding epitope (i.e. STD-TOCSY [55], STD-DOSY [56]).

For those sugar-binding receptors that are difficult to handle, STD-NMR *in vivo* (with living cells) might be a prospect of binder (and affinity) identification. In the last years, application of STD-NMR to living systems has allowed to circumvent the problem of receptor isolation (and purification). *In vivo* STD NMR experiments used to explore the interaction of DC-SIGN receptor against mannose-containing polysaccharides have opened the door to the use of this technique in a variety of experimental conditions. [57] For instance, the use of HR-MAS techniques together with STD NMR to avoid cell precipitation has improved and expanded the applicability of STD-NMR with whole cells.[58]

###### 3.3.2.2.1 WATERLOGSY (Water-Ligand Observed via Gradient SpectroscopY)

**Fig. (4):**
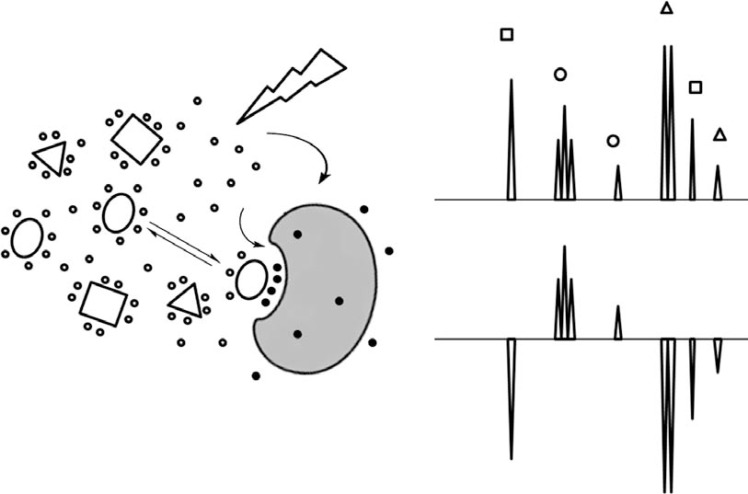
Schematic representation of the WATERLOGSY experiment.

This experiment is similar to STD, but magnetization is transferred from the bulk water to the free ligand via the protein-ligand complex. It has been successfully applied for ligand screening since binders and non-binders from a mixture of ligands can be easily discriminated as non-binder resonances have opposite sign (negative) and decreased intensity when compared with effective binders. [59] Ligand affinity can be estimated by performing the experiment in the absence and presence of the receptor and at different ligand concentrations (see Fig. **4**). Thus, the correlation between the intensity enhancement and ligand concentration allow for determination of dissociation constants. Alternatively, competition experiments can also be used in a qualitative way to probe binding events at the same binding site of known ligands. [60]

An interesting application of this technique has demonstrated the presence of structural water into the binding site of an antibody as a necessary bridge when a peptide-carbohydrate mimic occupies the binding pocket.[61] Determination of the specific location of these water molecules was further achieved through careful analysis of MD simulations. 

## 4 PROTEIN-CARBOHYDRATE INTERACTIONS IN HEALTH AND DISEASE

All cell surfaces are coated with complex carbohydrates acting as recognition molecules in host-pathogen interactions.[ 62] Protein-carbohydrate interactions mediate the first contact between the pathogen and the host, thus being involved in several disease states like inflammation, cancer, and infectious diseases. Understanding the bioactive conformation and the interactions between native carbohydrates and the corresponding receptor represents an important step towards the rational design of glycomimetics, which can exhibit improved drug-like properties such as increased affinity, stability, serum half-life, and bioavailability.[63] On the other hand, the molecular recognition study of carbohydrates at the microbial pathogens (bacteria and viruses) surface opens the door for developing carbohydrate-based vaccines educating our immune system to create antibodies.[64] NMR spectroscopy has revealed to be widespread useful to disclose interactions at the molecular level, namely in low affinity complexes (10^2^ - 10^6^ M^-1^) and when ligands are highly flexible, or even to support drug development and improvement. 

As mentioned above, pathogens such as bacteria carry complex carbohydrates like capsular polysaccharides. In this sense, NMR can be useful to investigate their conformational properties in solution and help to explain some biological events from the structural view point. For instance, the capsular polysaccharide of *Escherichia coli* K5 has been hypothesized to promote virulence through its molecular mimicry of the host heparan sulphate. To test this hypothesis, pure oligosaccharides from K5 capsular polysaccharide were synthesized and their conformational properties investigated with NMR.[65] This allowed for the full atomic assignment of the K5 hexasaccharide and revealed that all carbohydrate rings adopted a ^4^*C*_1_ conformation, where the amide side chains have a *trans* orientation and the hydroxymethyl group is freely exposed to the solvent. Initial models of the glycosidic linkage conformation based on interresidual NOEs suggest that the overall molecular geometries of K5 polysaccharide, heparan sulphate and even fully-sulphated heparin are remarkably similar. These results support the hypothesis that the K5 capsular polysaccharide confers virulence to *E. coli* K5 by being a 3D molecular mimetic of host heparan sulphate, helping it to evade detection by the mammalian immune system.

In addition, the recognition of *Candida albicans*, an opportunistic fungal pathogen which infects individuals with debilitated immune system, by host cells is directly mediated by the β-mannosylated epitopes that show a complex expression pattern on the *N*-glycan moiety of phosphopeptidomannans. Thus, delineation of the β-mannoside regulation and expression pathways has become an important milestone toward the comprehension of host-pathogen recognition. Using HR-MAS NMR methodology, Y. Guérardel and co-workers demonstrated the possibility of assessing the general profiles of cell-surface-exposed glycoconjugates from intact living yeast cells without any prior purification step.[66] This technique permitted to directly observe structural modifications of surface-expressed phosphodiester-linked β-mannosides on a series of deletion strains in β-mannosyltransferases and phospho-mannosyltransferases compared with their parental strains.

NMR has also been employed to characterize receptors found in the outer membrane of *Pseudomonas aeruginosa*. This is an opportunistic pathogen, which causes persisting life-threatening infections in cystic fibrosis patients and immunocompromised patients.[67] The impermeability of the *P. aeruginosa* outer membrane contributes substantially to the notorious antibiotic resistance of this human pathogen. In fact, it is accepted that this impermeability is partially provided by the outer membrane protein H (OprH). The structure of OprH in solution was recently solved by NMR in a membrane environment [68] and, through fast time-scale dynamics measurements, the authors showed that the extracellular loops of OprH were, in fact, disordered and unstructured. Furthermore, based on NMR chemical shift perturbations observed upon the addition of lipopolysaccharides (LPS) to OprH in lipid micelles, Tamm and collaborators demonstrated that LPS interacts directly with the outer membrane OprH via electrostatic interactions, but also through two exposed tyrosines in the middle of the bilayer. These results offer evidence for multiple interactions between OprH and LPS that could likely contribute to the antibiotic resistance of *P. aeruginosa*.

One important feature of protein-carbohydrates interactions in a host-pathogen interplay concerns to the fact that the sugar moieties on the bacterial cell surface often resemble those present on the host cell, so they can lead to the production of antibodies that cross-react with epitopes common to the pathogen and the host leading to autoimmune diseases.[ 69] Human natural killer-1 (HNK-1) is one of the most characteristic carbohydrate epitopes of the nervous system cell glycans and one of the targets of autoimmune antibodies, which lead to autoimmune-based peripheral neuropathies, like the Guillain-Barré syndrome.[70] The molecular recognition of HNK-1 trisaccharide and related oligosaccharides by Anti-HNK-1 antibodies, has been recently investigated by using SPR and STD-NMR experiments.[71] In order to assess the specificity of monoclonal HNK-1 antibodies, different HNK-1 carbohydrates were synthesized. SPR revealed the significance of the glucuronic acid in antibody recognition, while STD-NMR allowed disclosing the contribution of the different oligosaccharide moieties in the interaction HNK-1 pentasaccharide and the HNK-1 412 antibody. 

Also in this context, immune-mediated neuropathologies, such as the already mentioned Guillain-Barré, can occur as a result of the presence of auto-antibodies that target gangliosides, the glycosphingolipids predominantly found in the nervous system.[72] STD-NMR, in combination with SPR, allowed for the delineation of the molecular interaction between puriﬁed IgG auto-antibodies and gangliosides.[73] Since a large amount of pure antibody can be difﬁcult to obtain, STD-NMR also offers the possibility of direct detection of ganglioside-antibody interactions using untreated serum.[74] Thus, using sera samples of two patients, the authors demonstrated that the antibodies of one patient targeted two distinct gangliosides, GM1 and GM2, while the other patient sera only contained antibodies reactive against GM1. These results revealed that similar protocols could be eventually employed for diagnostic purposes.

STD-NMR was also used to investigate structural aspects of the interaction between a *Bacillus anthracis *tetrasaccharide and the monoclonal antibody (mAb) MTA1-3.[75] This tetrasaccharide contains three rhamnose residues and an atypical terminal saccharide, named anthrose, which after immunization of mice yielded the mAb MTA1-3.[76] STD results confirmed interaction of this tetrasaccharide with the antibody and pointed out that tight-binding sites were found within the β-anthrose(1➜3)Rha fragment.

The use of carbohydrates as vaccines has been limited for many years due to their weak immunogenic effects. Thus, one of the strategies for the design of carbohydrate-based vaccines is to use a carbohydrate-mimetic peptide as alternate ligand.[77] The *Shigella flexneri* Y strain has a cell-surface *O*-linked polysaccharide (LPS). Using phage display, a weakly immunogenic carbohydrate-mimetic peptide with the amino acid sequence MDWNMHAA, of the *S. flexneri* Y O-linked polysaccharide, was identified to be cross-reactive with the SYA/J6 monoclonal antibody [78] STD-NMR combined with CORCEMA-ST (COmplete Relaxation and Conformational Exchange Matrix Analysis of Saturation Transfer) analysis were used to probe the bioactive solution conformation of the carbohydrate mimic MDWNMHAA when bound to mAb SYA/J6.[79] 

Understanding receptor specificities at the atomic level can provide valuable information for the design of entry inhibitors against human pathogenic viruses establishing novel anti-viral therapies. In this context, noroviruses from the family of *Caliciviridae* are the major cause of non-bacterial acute gastroenteritis worldwide, imposing an important weight on health care systems.[80] At present, no direct treatment or vaccination strategy is available. STD-NMR has proved to be a valuable technique to detect and characterize the binding of small ligands and virus-like particles (VLP). As an example, Peters and co-workers unveiled the atomic details of the recognition of histo-blood group antigens (HBGAs) and fragments thereof by Calicivirus.[81] The experiments yielded binding epitopes of several HBGAs and showed that L-fucose and L-galactose represent the minimal structural requirements for specific molecular recognition by Calicivirus. STD-NMR studies of virus-ligand or VLP-ligand interactions benefit significantly from the large size of viruses or VLPs. However, careful precaution should be taking into account when setting the off- and on-resonance saturation frequencies and several controls should be performed to distinguish specific from non-specific binding. In the last year, the application of ligand-based NMR screening to identify compounds with virus-binding activity from a fragment library was reported.[82] Recently, the same team described the molecular details of the recognition of host cell blood group antigens by a human norovirus combining STD-NMR, trNOESY and molecular dynamics simulations.[83] L-fucose was identified as a minimal structural recognition element that is bound with remarkable specificity; while the neuraminic acid residue of sLe^x^ remains flexible in the bound state. This work opens the door for further systematic studies into norovirus-host carbohydrates recognition and delivers valuable details for the design of entry inhibitors.

A paradigmatic case of the study of host-pathogen recognition was recently reported, regarding the molecular basis of the relation between the blood group type and the severity of the El Tor Cholera infection.[84] Apparently, blood group O individuals are more severely affected by the El Tor biotype of *Vibrio cholerae* than those with blood groups A or B.[85] Curiously, there is no clear blood-group dependence for the *V. cholerae* classical biotype. The A, B and O blood groups are distinguished by carbohydrates present on the surface of cells.[86] The blood group O is characterized by oligosaccharides terminating in a 2-*O*-fucosyl-galactose structure while blood groups A and B are further substituted by an α-galactosamine or galactose moiety, respectively. Hence, to address this question, two monovalent blood group oligosaccharides were prepared: one mimicking the blood group B and another mimicking the blood group O. ITC and STD-NMR were used to evaluate the ability of the El Tor CTB from *V. cholerae* (CTB) and *E. coli* heat-labile toxin (LTBh) to bind to the blood group oligosaccharides mimics, A and O. STD results showed that the mimics of blood group O bind to both type of *V. cholerae* biotypes, while the mimics of blood group B only bind to LTBh biotype. Comparison of the LTBh and CTB structures led to propose that the blood group dependence of El Tor cholera could be attributed to a threonine-47-isoleucine mutation in the El Tor CTB protein. This mutation seems to prevent the El Tor CTB toxin from binding to blood group B oligosaccharides.

## 5 DRUG TARGETING OF PROTEIN-CARBOHYDRATE INTERACTIONS

As stated above, proteins and nucleic acids have been traditionally considered as the sole information-carrying molecules, but oligosaccharides of glycoconjugates exceed by far the storage capacity of those and might, therefore, be useful to design more effective target devices. The idea would be mimicking the body’s strategy to develop postal-code-like determinants.[87] When presented without a carrier, they have the tendency to saturate receptors on the cell surface and to block, for example, cellular interactions in anti-adhesion strategy. After carrier immobilization, avidity of the chosen determinant can be favorably increased. Also, further attachment of a therapeutically-active moiety and ensuing import of the cell-bound compound (targeting device and therapeutic agent) offer the potential to minimize undesired side effects by an accurate targeting process. 

### 5.1 The Concept of “Sugar Code”

**Fig. (5):**
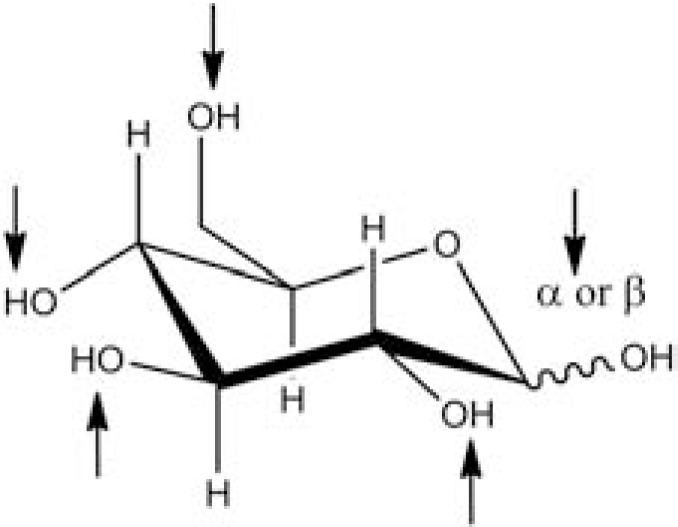
Glucopyranose structure. Positions that are susceptible for binding of other carbohydrates/substituents are marked with arrows.

The monosaccharides that commonly constitute glycoconjugates in higher organisms are glucose, mannose, galactose, N-acetylglucosamine, N-acetylgalactosamine, xylose and L-fucose, as neutral sugars, and glucuronic, galacturonic, xyluronic, L-iduronic and sialic acids. These would constitute the so-called “sugar alphabet”.[2,88,89] Carbohydrates constitute the most versatile molecules for the construction of a big set of oligo- to polymers, which is obvious by just looking at their structures (Fig. **5**). Each monosaccharide has a “glycosidic” hydroxyl group that takes part on forming oligosaccharides and additional hydroxyl groups that are as well available for subsequent modifications. Moreover, the glycosidic hydroxyl group can present two conformations, namely the α and β anomers.

Likewise, the rings of monosaccharides can show different sizes, i.e. six-membered pyranose and five-membered furanose rings. As well, an open form of the ring can be present in solution, although in a very small amount. Further modifications can be chemically produced over the oligosaccharide in several ways (e.g. acylation, sulfation, methylation, phosphorylation…).

All this together gives an idea of the enormous number of combinations that can lead to diversification of a saccharide chain being much higher than in the case of proteins or nucleic acids, thus supporting the possibilities of carbohydrates as coding structures and efficient means of biological information storage. A high density coding system is essential to allow cells to communicate efficiently through complex surface interactions.

### 5.2 Lectin-Mediated Drug Targeting

Sugar-binding proteins show very shallow and solvent exposed binding sites and, as a result, they make only few direct contacts with their target ligands Thus, a key phenomenon leading to the specificity of such interactions resides in “multivalency”, arising from multiple protein-carbohydrate interactions which cooperate in a recognition event to achieve the necessary functional affinity. This leads to the need of multiple receptors arranged in such a way to bind efficiently to multiple saccharide ligands.[89,90] Also, individual receptors can contain more than one binding site or oligomerize to form larger structures with multiple binding sites. The characteristics of binding can be tuned by alteration of the individual saccharide residues or their relative orientations, recognition events can be readily and flexibly modulated and kinetics of multipoint attachment will be different from those involved in single processes. The possibility to synthesize molecules composed by multiple carbohydrate residues would allow the identification of uncharacterized binding events as multivalent, modulation of carbohydrate-mediated cell or virus binding, targeting of conjugates to particular cell types, immobilization of specific cell types and inducement of cell responses through selective binding and/or aggregation of cell-surface receptors.

As noted in the introduction, lectins are the biggest family of proteins that recognize carbohydrates and they can be used as tools for the isolation, characterization and localization of glycoconjugates.

The information encoded by the carbohydrate (message) is processed by a complementary recognition step by the lectin (translator), which can then be followed by a variety of post-binding signaling processes. This constitutes the basis for lectin-mediated drug targeting.[87] Especially relevant is the assessment of cell surface expression of glycoligand binding sites. Once the specificity of binding is established, the molecular nature of the binding sites can be studied.

Characterization of sugar residues on cancer cells with lectins is used to diagnose cancer malignancy, which illustrates the potential of sugars as specific ligands for the specific delivery of drugs or genes. It is important to remark that the low specificity of sugar-lectin interactions could be a strong limitation to glycotargeting. Indeed, diversity of simple sugars is limited and lectins usually have large binding sites which can accommodate several simple sugars, being therefore able to recognize several glycoconjugates, thus showing little cell specificity.[91]

### 5.3 Examples of Drug Targeting Mediated by Protein-Carbohydrate Interactions

Several groups, including ours, are involved in the design and synthesis of glycomimetics by sca old replacement. One possibility we have proposed is the use of conformationally stable cyclic diols to replace non-pharmacophoric parts of bioactive oligosaccharides, while preserving the proper pharmacophore orientation. Computational tools have shown their capability for the prediction of three-dimensional structures of the mimics, allowing the comparison of the structure of the inhibitor with that of the natural ligand. When supported by adequate experimental work, molecular modeling also makes it possible to obtain at least qualitative predictions on the binding mode of new substrates, and to design further simpliﬁcations of the glycomimetic structures aimed at reducing the carbohydrate-likeness of the mimics and increasing their drug-like properties. This approach has been validated by S. Mari and coworkers [92] by using, as a model system, the recognition pair composed of the head-group of ganglioside GM1 and the two bacterial enterotoxins (cholera toxin (CT) and heat-labile toxin of E. coli (LT)) that target it on cell membranes. The new ligands were designed starting from well known GM1 mimics by replacement of their GalNAc residue with the C4 isomer GlcNAc. It is known that GM1 interacts with CT and LT using the Gal and NeuAc residues at the oligosaccharide non-reducing end. All mimics used in the study have been shown to interact with their target with the same affinity and no loss of cooperativity when the reducing end lactose is replaced by an appropriate diol. Sialic acid could also be replaced by different hydroxyacids but in this case affinity varies depending on the hydroxyacid and cooperativity is lost.

In a later work, [93] pseudo-GM1 ligands including a cyclohexyl group and a phenyl group were tested and showed a significant affinity for the cholera toxin. A detailed NMR study of the toxin/mimic complexes, assisted by molecular modeling techniques, allowed their interactions with the toxin to be explained at the atomic level. It was shown that intramolecular van der Waals and CH/π carbohydrate-aromatic interactions define the interaction of the mimic presenting a phenyl moiety which adopts a three-dimensional structure significantly more well-suited for proper interaction with the toxin. The exploitation of this kind of sugar-aromatic interaction, which is very well described in the context of carbohydrate/protein complexes, may open new avenues for the rational design of sugar mimics. Although the two ligands described in the mentioned work are similar in nature and activity, a detailed analysis of their behavior in solution and in the binding site of the cholera toxin reveals striking differences, in terms of both, conformational flexibility and binding mode. Intra- and intermolecular interactions among the different residues strongly modulate the conformational features of these molecules in solution and when bound to the toxin.

Rational drug design is normally predicated on knowledge of the three-dimensional structure of the protein-ligand complex and the thermodynamics of ligand binding but, despite the fundamental importance of both enthalpy and entropy in driving ligand binding, the role of conformational entropy is rarely addressed in drug design. The role of conformational entropy and its relative contribution to the free energy of ligand binding to the carbohydrate recognition domain of galectin-3 has been proved.[94] Using a combination of NMR spectroscopy, isothermal titration calorimetry, and X-ray crystallography, the binding of three ligands with dissociation constants ranging over 2 orders of magnitude was studied. ^15^N and ^1^H spin relaxation measurements showed that the protein backbone and side chains respond to ligand binding by increased conformational ﬂuctuations, on average, that differ among the three ligand-bound states. Variability in the response to ligand binding is prominent in the hydrophobic core, where a distal cluster of methyl groups becomes more rigid, whereas methyl groups closer to the binding site become more ﬂexible. The results reveal an intricate interplay between structure and conformational ﬂuctuations in the different complexes that ﬁne-tunes the afﬁnity. The estimated change in conformational entropy is comparable in magnitude to the binding enthalpy, demonstrating that it contributes favorably and signiﬁcantly to ligand binding. The authors speculate that the relatively weak inherent protein-carbohydrate interactions and limited hydrophobic effect associated with oligosaccharide binding might have exerted evolutionary pressure on carbohydrate-binding proteins to increase the afﬁnity by means of conformational entropy.

The accurate intra- and inter cellular routing of macromolecules sets an example for the development of carrier systems for targeted drug delivery. By making use of the natural postal-code-like molecular determinants, cell-type-specific uptake of drugs can then be achieved. Besides peptide motifs, the discovery of galactose/N-acetylgalactosamine-dependent hepatic clearance of serum glycoproteins has spurred to explore the applicability of oligosaccharides as targeting devices. Synthetic mono-, bi-, and trivalent galactosides and lactosides were prepared with two different spacer lengths and it was tested whether and to what extent they interfered with galectin binding to a surface-immobilized neoglycoprotein or asialofetuin and to cell surfaces.[ 95] The possibility of having one binding site per galectin molecule (galectin-3) or two carbohydrate recognition domains at opposing sides of a homodimeric molecule (galectin-1) was examined. For further comparison, a plant lectin with the same monosaccharide specificity and a lactoside-binding immunoglobulin G fraction from human serum were included. The expectable ranges of reactivity of the synthetic glycosides for C-type lectins and galectins are defined. The abundance and spatial accessibility of β-galactosides renders it likely that such determinants are docking points for more than one class of endogenous lectins. It is thus not only important to tailor properties with exclusive consideration of the target lectin in focus. Additionally, the extent of interference of the applied glycoside system with other lectins is to be carefully examined to preclude clinical side effects. Similar to C-type lectins, the length of the aglyconic spacer can significantly modulate ligand performance. The presence of galactose as sugar ligand is inferior to its extension by a glucose residue for galectins in contrast to the C-type lectins. Branching does not generate a remarkable glycoside cluster effect for galectins. Similar to galectins, the binding of the immunoglobulin G fraction was primarily sensitive to the lactose-containing triglycoside. An indication for a significant side effect by the trigalactoside on this receptor system could not be delineated.

Carbohydrates not only represent a vast potential as structure building blocks of living cells, but also have proved their worth as a promising candidate for drug delivery.[96] Glycosylation of nanocarriers leads to the evolution of promising delivery systems. Some advantages of glycosylated carriers include the release proﬁle of bioactives when introduced into a biological system. Being natural products of living systems, these carriers also constitute multifaceted drug delivery vehicles and reduce the toxicity associated with the unmodiﬁed drug carrier and therapeutic agent. An additional attribute of these carriers is to positively alter the kinetic proﬁle of drugs via its stabilization. The presence of lectin receptors on different cell surfaces makes the glycosylated carrier appreciable for targeted delivery of drugs to improve their therapeutic index. Active participation of a number of lectin receptors in immune responses to antigens overlaid the application of glycosylated carriers to antigen delivery and immunotherapy for treatment of ailments like cancer. These advantages revealed the promising potential of glycosylated carriers in each perspective of drug delivery. 

Proposals to target glucose metabolism as a selective mechanism by which to kill cancer cells has been recently reviewed.[97] Cellular transformation is associated with the reprogramming of cellular pathways that control proliferation, survival, and metabolism. Among the metabolic changes exhibited by tumor cells, an increase in glucose metabolism and glucose dependence is present. Targeting metabolic pathways for cancer therapy seems appealing at first glance, as enzymes are attractive molecular targets. However, to be an attractive candidate for cancer therapy, there must be a significant difference in the requirement for a given enzyme’s activity between cancer cells and normal proliferating cells. A few potential candidates that show overexpression in certain cancer lines have been identified, including GLUT1, HKII, PHGDH, and LDH-A.

As we have recently reviewed, [98] certain oligosaccharides are characteristically expressed on cancerous cells and represent excellent targets for anticancer vaccines or drugs. However, the successful use of carbohydrates as vaccines has been limited for many years due to the weak immunogenic effects, usually restricted to IgM antibody response, particularly a T-independent cell response without memory function, and poor response in infants. An efficient strategy to overcome these problems has focused on the design of glycoconjugate vaccines, in which an appropriate glycan is covalently attached to a carrier protein or peptide. This approach has proved to be the most effective, since it may induce T-cell responses giving a protective immunity against a wide range of bacterial pathogens, which express a glycocalyx and where antibodies directed against cell surface saccharides are protective. An alternative strategy consists in the use of peptides as sugar mimetics either to replace the carbohydrate molecule as a vaccine, or to supplement it in order to boost the immune response. The study of how complex carbohydrates interact with antibodies represents an important step toward the design of carbohydrate-based vaccines. In this context, the mentioned review focuses on the application of NMR methods to characterize the main structural features that govern the recognition of carbohydrates by antibodies.

Hyaluronic acid-L-cysteine conjugate was synthesized and characterized for mucoadhensive drug delivery.[99] Adhesion studies on the mucosa revealed an increase in the adhesion force of the obtained versus unmodified hyaluronic acid. The inhibitory effect of hyaluronic acid toward trypsin and chymotrypsin was significantly improved compared to hyaluronic acid. Permeation studies utilizing a MDCK cell monolayer system demonstrated a sustained drug release. Based on these features the novel thiolated polymer might represent a promising multifunctional excipient for various drug delivery systems.

## 6 CONCLUSION

The increasing interest in carbohydrates as key information carriers involved in central biological processes has prompted the investigation of their multiple facets from different viewpoints. The knowledge on the structural aspects governing the molecular recognition between sugars and lectins gained through NMR and molecular modeling has been successfully translated into emerging biomedical strategies, such as the use of carbohydrates as delivery vehicles of pharmaceuticals and the design of lectin inhibitors. The increasing realization of the importance of glycans in physiological and pathological phenomena, backed by the virtues of NMR for the scope of sugar-protein interactions are likely expanding these applications further in the near future.
